# Evaluation of Ongoing Crisis Intervention Team (CIT) Training for Law Enforcement Using the ECHO Model

**DOI:** 10.1007/s11896-022-09529-3

**Published:** 2022-06-17

**Authors:** Annette S. Crisanti, Jaymes Fairfax-Columbo, Danielle Duran, Nils A. Rosenbaum, Ben Melendrez, Isaac Trujillo, Jennifer A. Earheart, Matthew Tinney

**Affiliations:** 1 Department of Psychiatry and Behavioral Sciences, University of New Mexico, MSC 09 5030, Albuquerque, NM 87131 USA; 2 Better Statistical Consulting, Albuquerque, USA; 3 Behavioral Health Division, Albuquerque Police Department, Albuquerque, USA; 4 Crisis Intervention Unit, Albuquerque Police Department, Albuquerque, USA

**Keywords:** CIT training, ECHO, Law Enforcement Officer training, CIT best practices

## Abstract

Crisis Intervention Team (CIT) training aims to improve law enforcement officers’ (LEOs) ability to safely intervene in calls for service involving individuals with mental illness, as well as to increase LEOs’ ability to link these individuals to mental health services and divert them from the criminal justice system. However, most CIT training is delivered as a stand-alone class, and continuing education in CIT principles and best practices is limited. To address this problem, the Albuquerque Police Department, in partnership with the Department of Psychiatry and Behavioral Sciences at the University of New Mexico, developed CIT ECHO to provide continuing education in CIT best practices. The authors evaluated 113 weekly CIT ECHO sessions targeting LEOs in New Mexico, offered between 2017 and 2020. LEOs electronically completed a post-session survey after each didactic; additionally, a targeted follow-up survey was distributed to LEOs participating in at least three sessions. Surveys measured impact of CIT ECHO on knowledge, self-efficacy, and attitudes towards individuals with mental illness involved in the criminal justice system. After participating in CIT ECHO, LEOs reported increases in knowledge of didactic content and that they felt comfortable applying didactic content on the job. LEOs also evidenced positive attitudinal shifts towards individuals with mental illness and criminal justice involvement. Continuing education in CIT best practices appears to increase LEOs’ knowledge base and comfort in working with individuals with mental illness and criminal justice involvement, as well as results in positive attitudinal shifts towards this population.

## Introduction

The Crisis Intervention Team (CIT) model is one of the most universally accepted mental health (including substance use) training programs in the country for law enforcement officers (LEOs) (Compton et al. 2011). The 40-h CIT class is often referred to as the gold standard for training LEOs in understanding mental health and addictions (Compton et al. 2008; Watson et al. 2010; Watson and Wood 2017). CIT aims to improve officers’ ability to safely intervene in calls for service involving individuals with mental illness and to increase officers’ ability to link individuals to mental health services and divert them from the criminal justice system when appropriate. CIT training has been associated with improved knowledge, attitudes, and self-efficacy among LEOs and improved outcomes between law enforcement and people living with mental illness (Bahora et al. 2008; Bonfine et al. 2014; Compton et al. 2006, 2011; Ellis 2014; Skeem and Bibeau 2008; Teller et al. 2006; Watson et al. 2010).

CIT training is a stand-alone class of up to 40 h provided by police trainers, mental health clinicians, consumers, and family advocates. The curriculum includes training on signs and symptoms of mental illness, co-occurring disorders, mental health treatment, and de-escalation techniques (Watson and Fulambarker 2012). A limitation of the CIT model is that it is typically a one-time course, and opportunities for continuing education in CIT principles and best practices are variable and limited (Plotkin and Peckerman, 2017). Furthermore, research suggests that mental health-related training for LEOs tends to be frontloaded, with a substantially greater focus placed on entry-level mental health training as opposed to in-service training (Fiske et al. 2020; Plotkin and Peckerman 2017). This is problematic, as research suggests that knowledge gains from CIT generally diminish over time, prompting recommendations that CIT officers may benefit from continuing education efforts regarding mental health topics (Compton and Chien 2008). To this end, some empirical basis exists for provision of CIT training over time producing similarly positive outcomes to provision of CIT as a one-time training (Cuddeback et al. 2016).

To address the need for continuing education in CIT best practices, the Albuquerque Police Department, in partnership with the Department of Psychiatry and Behavioral Sciences, at the University of New Mexico (UNM), developed CIT ECHO (Extension for Community Healthcare Outcomes) (Crisanti et al. 2019). The mission of CIT ECHO is to improve law enforcement interactions with people with a mental illness and with the aim of increasing the safety of law enforcement and individuals in mental health crisis, fostering connections with the mental health system, destigmatizing mental illness for law enforcement, and raising the level of community policing. CIT ECHO connects LEOs throughout New Mexico and the country via videoconferencing to a central hub of CIT experts and psychiatrists. More specifically, the hub team includes two psychiatrists (one from the Albuquerque Police Department and one from the UNM Department of Psychiatry and Behavioral Sciences), a CIT detective, a crisis specialist, and a project coordinator. A unique component of CIT ECHO is that it is based on Project ECHO®, an evidence-based videoconferencing platform originally designed to link primary care physicians to a network of healthcare specialists to receive ongoing mentoring and feedback on complex patient cases (Arora et al. 2011). While the ECHO model has been documented as an effective tool for continuing medical education in several subspecialty areas (Arora et al. 2017; Komaromy et al. 2017), it had not previously been used with law enforcement prior to CIT ECHO. CIT ECHO provides a weekly opportunity for LEOs to receive CIT-related training and to review complex cases with the CIT ECHO hub team and other LEOs in the network. CIT ECHO provides an opportunity for all LEOs to present cases they are working on and then learn by discussing these cases with the group; LEOs receive responses to specific questions from psychiatrists in real-time. The use of videoconferencing allows participants (mostly law enforcement officers) to connect to CIT ECHO through their smartphones, laptops, and patrol vehicle computers.

Details regarding the implementation of CIT ECHO, including challenges and its impact on law enforcement practices, were previously described by Crisanti et al. (2019). The purpose of this article is to describe the results from the evaluation of CIT ECHO. We describe the impact of CIT ECHO on (1) knowledge related to CIT best practices, (2) self-efficacy with respect to interactions with individuals with mental illness, and (3) attitudes towards mental illness among individuals involved in the criminal justice system. More specifically, we were interested in learning whether CIT ECHO increased knowledge of CIT best practices and self-efficacy, and improved attitudes towards people living with mental illness involved in the criminal justice system.

## Methods

### Program Description

CIT ECHO sessions were held weekly for 90 min and, following the ECHO model, consisted of three main parts: (1) a didactic presentation related to CIT policing or mental health; (2) a case debriefing involving mental health and/or substance use, presented by officers; and (3) interactive discussion between the detectives and psychiatrists on the hub team and the participants of the material covered during the didactic and the case presentation. Each component lasted approximately 30 min. The program coordinator was responsible for scheduling presenters for the didactics and categorizing each didactic into one of seven key topic areas (hereafter referred to as modules), including (1) CIT Policing, (2) Resources, (3) Psychiatric Diagnosis, (4) De-escalation and Communication Skills, (5) Officer Self-Care, (6) Substance Use, and (7) Special Training. A unique component of CIT ECHO is that the topics for the didactics are driven by officer input in conjunction with the CIT ECHO hub team. Didactic topics must align within one of the seven modules. All CIT ECHO sessions were conducted via Zoom, a videoconferencing platform. During the study period, the target population for CIT ECHO was LEOs in New Mexico (primarily in Bernalillo County), including uniformed and non-uniformed officers, with the latter typically being detectives. Although LEOs from other states also attended CIT ECHO, as well as other public safety personnel (e.g., corrections officer), mental health professionals, and psychiatry residents, this evaluation was restricted to uniformed or non-uniformed LEOs in New Mexico.

### Procedure

Two surveys, referred to as the post-session survey and the follow-up survey, were developed by the CIT ECHO hub team to evaluate the impact of CIT ECHO. The post-session survey was intended to be completed by all participants following the end of a CIT ECHO session. The follow-up survey was restricted to LEOs who completed three or more CIT ECHO sessions. Both surveys were administered electronically using REDCap (Research Electronic Data Capture), which is a secure web application for building and managing online surveys and databases. After each session, the CIT ECHO program coordinator emailed a link to the post-session survey to every participant. Participants had 1 week to complete the post-session survey. Post-session survey data were collected from June 2018 to December 2020. With respect to the follow-up survey, the CIT ECHO program coordinator identified LEOs who completed three or more CIT ECHO sessions on a weekly basis and emailed a link to the electronic follow-up survey. Officers had 2 weeks to complete the survey, with one reminder sent 1 week following the initial email. Follow-up evaluation data were collected during April 2017–September 2018.

Both surveys collected basic demographic data, including gender, whether LEOs were uniformed or non-uniformed (referred to as position), and years worked in public safety, with response categories for this last variable being less than 3 years, 3–9 years, 10–15 years, and greater than 15 years. Attendance for each session was tracked using iECHO, a web-based proprietary program management software and database developed and managed by the ECHO Institute at the University of New Mexico. This evaluation was approved by the University of New Mexico, Human Research Protections Office, Institutional Review Board (protocol number: 16-429). All participants provided passive consent prior to completing the surveys.

### Measurement of Outcomes

#### Knowledge

The impact of CIT ECHO on knowledge was assessed by two questions included in the post-session survey that asked “To what extent did the information in this week’s session improve your knowledge of the presentation topic?” and “To what degree did this week’s session impact your knowledge of resources in the community?” Participants were asked to rate their level of agreement using a 5-point Likert scale ranging from 1 = not at all to 5 = very much.

### Self-Efficacy

The impact of CIT ECHO on perceptions of self-efficacy related to interacting with individuals with mental illness was assessed by three questions in the post-session survey and four statements in the follow-up survey. The first question in the post-session survey asked “How likely are you to use the information that you learned in this week’s session in your job?” Participants were asked to rate their level of agreement using a 5-point Likert scale ranging from 1 = definitely not to 5 = definitely will. The second question in the post-session survey asked “After attending this weeks’ session, how would you now rate your ability to interact with an individual in a mental health crisis?” Participants were asked to rate their level of agreement using a 5-point Likert-type scale ranging from 1= poor to 5 = excellent. The third question in the post-session survey required a yes/no response, and asked “Based on the information you received in this week’s session are there any changes that you would make in your interactions with people living with mental illness?” The four self-efficacy-related statements in the follow-up survey required participants to rate their level of agreement using a 5-point Likert scale ranging from 1 = strongly disagree to 5 = strongly agree. The four statements were, because of CIT ECHO, (1) “I feel comfortable interacting with people living with a mental illness”; (2) “I feel I am able to determine if a person living with a mental illness, who has committed a crime, should be taken to jail or to a hospital/emergency room”; (3) “I am able to utilize verbal de-escalation techniques effectively”; and (4) “I know who to call for advice about how to interact with a person living with a mental illness.”

### Attitudes

The impact of CIT ECHO on attitudes towards individuals with mental illness involved in the criminal justice system was assessed in the follow-up survey using a retrospective pre-post design (Bhanji et al. 2012; Geldhof et al. 2018; Thomas et al. 2019). In this design, both before and after information was collected contemporaneously. Participants who completed three or more CIT ECHO sessions were first asked to reflect back and indicate how they would rate their level of agreement to six statements before participating in CIT ECHO sessions. They were also asked to think about how they would rate their level of agreement to the same six statements now that they had participated in three or more CIT ECHO sessions. Respondents were asked to rate their responses to the six statements using a 5-point Likert scale ranging from 1 = strongly disagree to 5 = strongly agree*.* The six retrospective pre-post statements were (1) “People living with a mental illness are more likely to be the victim of a crime than to commit a crime,” (2) “People living with a mental illness are more likely to be the victim of violence than to be violent to others,” (3) “The symptoms of mental illness get better with treatment,” (4) “It is the job of law enforcement to link/connect people living with a mental illness into treatment,” (5) “People living with mental illness respond to de-escalation techniques,” and (6) “People living with mental illness do not always require the use of force to maintain officer safety.” Two total “attitude scores” were generated by summing up the first and second set of responses. While not a “genuine” or traditional measure of attitudes prior to attending CIT ECHO, a retrospective post-then-pre design has been determined to be a valid and reliable way to evaluate changes in attitudes over time (Bhanji et al. 2012; Thomas et al. 2019). Compared to a traditional pre-post design, the retrospective pre-post design is more convenient, takes considerably less time and resources, and is more likely to reduce response shift bias (Kaushal 2016).

### Statistical Analysis

Survey responses were first examined for missingness. Multiple imputation was used to impute plausible values for those instances in which data were missing, using an approximate Bayesian framework with customization of a conditional model.

### Post-Session Survey

To account for the repeated surveys recorded by multiple individuals on post-session surveys (and unequal sample sizes therein), nonlinear mixed-effects models (NLME) were used to examine demographic and module effects. Individually, demographic and module effects were examined in the fixed portion of the model, while a random effect on unique participants controlled for within-subject variation (applied to all models). Likert-type responses were treated as continuous on the post-session survey. For the purposes of interpretability, estimates and 95% confidence intervals produced directly from the NLME models are presented; *p*-values for module or demographics (when applicable) were extracted from an analysis of variance of the NLME models.

### Follow-Up Survey

The follow-up survey contained reverse-coded Likert scale questions, so quality checks for straight-line answers were performed prior to formal statistical analysis. Three records were found to be straight-line answers across all Likert-type questions. To address this, the reverse-coded questions were marked as missing and imputed during the missing data step. We also checked for data missing patterns and found that data were missing completely at random. An overall index of attitudinal shift both before and after participation in CIT ECHO was constructed by summing responses to the six retrospective pre- and post-statements. Bivariate analyses between demographic characteristics and self-efficacy questions were conducted with chi-square tests, unless actual cell counts were less than expected cell counts, in which case Fisher’s Exact Test was implemented (no test statistic is reported for Fisher’s exact test). For bivariate analyses, Likert-type responses were recoded into three groups: disagree, neutral, and agree. The Wilcoxon signed-rank test was used to examine change in the overall attitude scores, pre- to post-attendance. Finally, principal component analysis (PCA), a data dimensionality reduction technique, was applied to the six attitude statements to increase interpretability and extract the dominant sentiments of attendees, pre- and post-CIT ECHO attendance. Principal components were examined for significant changes pre- to post-survey via the Mann-Whitney *U*-test, and differences by position and years of service via the Kruskal-Wallis test. All analyses were conducted using RStudio for Mac, version 1.4.1106.

## Results

There were 113 CIT ECHO sessions during the evaluation window for the post-session survey. With respect to the focus of the didactics, 16 (14%) focused on CIT Policing, 9 (8%) focused on Resources, 35 (31%) focused on Psychiatric Diagnosis, 8 (7%) focused on De-escalation and Communication Skills, 15 (13%) focused on Officer Self-Care, 12 (11%) focused on Substance Use, and 18 (16%) focused on Special Training.

### Sample Demographics

A total of 598 post-session surveys were completed (response rate 18.5%). After filtering out substantially incomplete records (i.e., those with only demographic data or the survey open timestamp, *n =* 53), 545 records remained, representing 120 unique individuals. 72.5% male (*n* = 87) and 78.3% were uniformed law enforcement (*n* = 94). Twenty-five percent of participants had been in the field for 3 years or fewer (*n* = 30); 19.2% between 4 and 9 years (*n* = 23); 20% between 10 and 15 years (*n* = 24); and 35.8% for more than 15 years (*n* = 43). The median number of sessions attended was one, with an interquartile range of one to four sessions and a range of one–55 sessions. A total of 59 follow-up surveys were completed (response rate = 48.7%); however, one record was removed for substantial incompleteness, resulting in a final dataset of *n* = 58. 76% were male (*n* = 44) and 67% were uniformed (*n* = 39). Fourteen percent of participants had been in the field for 3 years or fewer (*n* = 8); 15.8% between 4 and 9 years (*n* = 9); 40.4% between 10 and 15 years (*n* = 23); and 29.8% for more than 15 years (*n* = 17).

### Impact of CIT ECHO on Knowledge

*Results from the Post-Session Survey:* Module topic significantly impacted CIT ECHO participants’ knowledge of the information presented in the weekly sessions (*F* = 2.553; *p*=0.019). As shown in Table 1 (where *β* values correspond to the Likert scale anchors used for each question), the modules Psychiatric Diagnosis (*β* = 4.18) and Substance Use (*β* = 4.04) significantly increased participant knowledge, compared to other modules. The module topic also significantly impacted participants’ knowledge of resources in the community (*F* = 4.222; *p* < 0.001). More specifically, the modules Resources (*β* = 3.89), Substance Use (*β* = 3.73), Psychiatric Diagnosis (*β* = 3.72), and CIT Policing (*β* = 3.6) were significantly more helpful than other modules on increasing knowledge of other resources in the community. Length of service and position did not significantly influence responses.

**Table 1:** Impact of CIT ECHO module topic on knowledge and self-efficacy towards people with mental illness involved in the criminal justice system

	Module	*β*^1^	95% confidence interval	*F*_df1, df2_	*p*-value
***Knowledge***					
*To what extent did the information in this week’s session improve your knowledge of the presentation topic?*	CIT Policing	3.92	(3.74, 4.09)	2.55_1,6_	0.019
	De-escalation/Communication Skills	4.00	(3.84, 4.32)		
	Psychiatric Diagnoses	4.18	(4.09, 4.61)		
	Resources	3.86	(3.53, 4.07)		
	Officer Self-Care	3.97	(3.83, 4.23)		
	Special Training	3.88	(3.63, 4.07)		
	Substance Use	4.04	(3.95, 4.39)		
*To what degree did this week’s session impact your knowledge of resources in the community?*	CIT Policing	3.60	(3.39, 3.82)	4.22_1,6_	<0.001
	De-escalation/Communication Skills	3.71	(3.54, 4.09)		
	Psychiatric Diagnoses	3.72	(3.64, 4.04)		
	Resources	3.89	(3.88, 4.49)		
	Officer Self-Care	3.35	(2.86, 3.32)		
	Special Training	3.40	(2.95, 3.45)		
	Substance Use	3.73	(3.60, 4.10)		
***Self-Efficacy***					
*How likely are you to use the information that you learned in this week’s session in your job?*	CIT Policing	3.89	(3.74, 4.09)	4.59_1,6_	<0.001
	De-escalation/Communication Skills	4.11	(3.95, 4.43)		
	Psychiatric Diagnoses	4.08	(4.16, 4.52)		
	Resources	3.82	(3.49, 4.03)		
	Officer Self-Care	3.84	(3.40, 4.10)		
	Special Training	3.61	(3.36, 3.79)		
	Substance Use	4.03	(3.94, 4.38)		
*After attending this week’s session, how would you now rate your ability to interact with an individual in a mental health crisis?*	CIT Policing	3.64	(3.47, 3.81)	4.21_1,6_	0.027
	De-escalation/Communication Skills	3.65	(3.46, 3.87)		
	Psychiatric Diagnoses	3.80	(3.80, 4.11)		
	Resources	3.51	(3.16, 3.61)		
	Officer Self-Care	3.58	(3.35, 3.69)		
	Special Training	3.58	(3.34, 3.71)		
	Substance Use	3.66	(3.50, 3.87)		

^1^*β* corresponds to values on the Likert scales. The Likert scale for the knowledge questions ranged from “1” not at all, “2” very little, “3” somewhat, “4” much, “5” very much. The Likert scale for the first self-efficacy questions ranged from “1” definitely not, “2” probably not, “3” possibly, “4” probably will, “5” definitely will. The Likert scale for the second self-efficacy questions ranged from “1” poor, “2” fair, “3” average, “4” good, “5” excellent

### Impact of CIT ECHO on Self-Efficacy

#### Results from Post-Session Survey

The module topic significantly impacted responses to both statements around self-efficacy (Table 1). Module topic significantly impacted the likelihood that CIT ECHO participants would use the information presented in the weekly sessions on the job (*F* = 4.5933; *p* < 0.001). Specifically, the modules De-escalation and Communication Skills (*β* = 4.11), Psychiatric Diagnosis (*β* = 4.08), and Substance Use (*β* = 4.03) significantly increased the participant likelihood of utilizing information learned during that week’s session on the job, compared to other modules. Similarly, participants reported that module topic significantly impacted their ability to interact with an individual in a mental health crisis (*F* = 2.406; *p* = 0.027). The module Psychiatric Diagnosis (*β* = 3.80) significantly increased participant confidence, compared to other modules. Length of service and position did not significantly influence responses

Participants were also asked, “Based on the information you received in this week’s session are there any changes that you would make in your interactions with people living with mental illness?” Individuals were 58% less likely to respond “yes” to this question when the module was Self-Care and 70% less likely to respond “yes” when the module was Special Training (*p* = 0.047 and *p* = 0.026, respectively). Although not statistically significant, respondents were 20% more likely to respond “yes” when the module topic was De-escalation/Communication Skills or Psychiatric Diagnosis.

### Results from Follow-Up Survey

The follow-up survey showed that 89.7% (*n* = 52) of participants agreed that they felt comfortable interacting with people living with mental illness after participating in CIT ECHO. Forty-eight percent (*n* = 28) of participants agreed that they were able to determine if a person living with mental illness should be taken to jail or to a hospital/emergency room because of their participation in CIT ECHO. Fifty-seven percent (*n* = 33) of participants agreed that due to the CIT ECHO sessions, they were now able to utilize verbal de-escalation techniques effectively. Eighty-four percent (*n* = 49) agreed that they know who to call for advice about how to interact with a person living with a mental illness.

Significant associations were found between position and two of the four statements that assessed self-efficacy on the follow-up survey. Uniformed LEOs were significantly more likely to agree that they are able to determine if a person living with mental illness should be taken to jail or to a hospital/emergency room, compared to non-uniformed LEOs (62% compared to 47%, *p* = 0.042). Uniformed LEOs were also significantly more likely to agree with the statement, “I am able to utilize verbal de-escalation techniques effectively” compared to non-uniformed LEOs (90% compared to 74%, *p* < 0.001).

### Impact of CIT ECHO on Attitudes

#### Results from the Follow-Up Survey

Table 2 summarizes the responses to the pre-/post-retrospective statements. Responses shifted significantly towards “Agree” on all pre-/post-retrospective questions. Considering pre- and post-time points separately, significant differences were found by position on the statement, “People living with a mental illness are more likely to be the victim of a crime than to commit a crime” after attending CIT ECHO training sessions. The specific difference was found between “disagree” and “neutral” responses. Significantly more uniformed LEOs responded “neutral” to this statement than non-uniformed LEOs, after attending CIT ECHO sessions (21.05% vs. 0%, respectively; *p* = 0.004); however, when considering the pre-post time component differences between position became non-significant. The median response on the overall attitudinal index shifted significantly from pre to post: the median response at post was “agree,” or a score of 24, whereas the median response pre-CIT ECHO was “neutral,” or 19 (*W* = 751.5, *p* < 0.001). No significant differences were detected by position or length of service when considering demographic characteristics and time point (pre/post) together, for any of the individual attitudinal statements or the overall index.

**Table 2:** Impact of CIT ECHO on attitudes towards people with mental illness involved in the criminal justice system: retrospective pre- and post-test scores

		Pre-test % (*N*)	Post-test % (*N*)	*p* value
*1. People living with a mental illness are more likely to be the victim of a crime than to commit a crime*	Agree Neutral Disagree	44.8 (26) 39.7 (23) 15.5 (9)	71 (40) 14 (8) 14 (8)	0.005
*2. People living with a mental illness are more likely to be the victim of violence than to be violent to others*	Agree Neutral Disagree	39.7 (23) 36.2 (21) 24.1 (14)	66.7 (38) 21.1 (12) 12.3 (7)	0.003
*3. The symptoms of mental illness get better with treatment*	Agree Neutral Disagree	67.2 (39) 19.0 (11) 13.8 (8)	80.7 (46) 12.3 (7) 7.0 (4)	0.049
*4. It is the job of law enforcement to link/connect people living with a mental illness into treatment*	Agree Neutral Disagree	34.5 (20) 29.3 (17) 36.2 (21)	68 (39) 11 (6) 21 (12)	< 0.001
*5. People living with mental illness respond to de-escalation techniques*	Agree Neutral Disagree	56.9 (33) 25.9 (15) 17.2 (10)	73 (41) 5 (3) 21 (12)	0.002
*6. People living with mental illness do not always require the use of force to maintain officer safety*	Agree Neutral Disagree	55.2 (32) 25.9 (15) 19 (11)	75 (43) 19 (11) 5 (3)	0.008
*Total median attitude score and interquartile range*		19 (17**–**26)	24 (21**–**26)	< 0.001

The PCA conducted on attitudinal statements resulted in a two-component solution for both pre- and post-CIT ECHO. Prior to CIT ECHO, the first two components explained 63% of the total variance (proportion of cumulative variance explained = 0.63); after CIT ECHO participation, the total variance explained by just two principal components increased to 71% (proportion of cumulative variance explained = 0.71). The individual statements most correlated with each component at each time point varied. Table 3 summarizes the correlations between statements and the principal components as well as the importance of each statement in each principal component. The primary sentiment (PC1) pre-CIT ECHO was that (1) “It is the job of law enforcement to link/connect people living with mental illness into treatment” (*r* = 0.79); (2) “People living with mental illness often do not require the use of force to maintain officer safety” (*r* = 0.78), and (3) “People living with a mental illness are more likely to be the victim of violence than to be violent to others” (*r* = 0.69). The secondary sentiment (PC2) pre-CIT ECHO was disagreement with the statement that “People living with a mental illness are more likely to be a victim of a crime than to commit a crime” (*r* = −0.77) and agreement with the statement “People living with mental illness respond to de-escalation techniques” (*r* = 0.55). Post-CIT ECHO, the primary sentiment (PC1) included the three statements that were found to be correlated with PCI pre-CIT ECHO, plus an additional statement that was correlated with PC2 pre-CIT ECHO. The additional statement is “People living with a mental illness are more likely to be a victim of a crime than to commit a crime.” Noteworthy is that pre-CIT ECHO participants disagreed with this statement, and it surfaced as a secondary sentiment. Post-CIT ECHO, participants’ attitudes shifted in that they agreed with this statement, and it became a primary sentiment (*r =* 0.85). The secondary sentiment post-CIT ECHO was that “People living with mental illness respond to de-escalation techniques” (*r* = 0.97) (which increased from *r =* 0.55 PC2 pre-CIT ECHO). The PCA results did not vary by field or years of service, indicating that all participants experienced a similar shift in attitudes.

**Table 3:** Correlation between and variance explained by CIT ECHO attitudinal statements and principal components, pre and post

	**PCA: pre**		**PCA: post**	
**Item**	**PC1: C.I (C.c)**	**PC2: C.I (C.c)**	**PC1: C.I (C.c)**	**PC2: C.I (C.c)**
*1. People living with a mental illness are more likely to be the victim of a crime than to commit a crime.*	0.55 (0.32)	**−0.77 (−0.69)**	**0.85 (0.52)**	**−**0.21 (**−**0.19)
*2. People living with a mental illness are more likely to be the victim of violence than to be violent to others*	**0.69 (0.39)**	**−**0.5 (**−**0.42)	**0.79 (0.43)**	**−**0.22 (**−**0.14)
*3. The symptoms of mental illness get better with treatment*	0.55 (0.28)	0.26 (0.14)	0.56 (0.31)	0.32 (0.19)
*4. It is the job of law enforcement to link/connect people living with mental illness into treatment*	**0.79 (0.54)**	0.19 (0.12)	**0.79 (0.55)**	0.13 (0.03)
*5. People living with mental illness respond to de-escalation techniques*	0.51 (0.31)	**0.55 (0.51)**	0.01 (0.03)	**0.97 (0.95)**
*6. People living with mental illness often do not require the use of force to maintain officer safety*	**0.78 (0.53)**	0.16 (0.23)	**0.63 (0.95)**	0.11 (0.11)
Proportion of Variance	0.43	0.2	0.43	0.28
Cumulative of Variance	0.43	0.63	0.43	0.71

Bolded values represent strong to very strong correlations

*C.I.* component loadings (correlation between principal component and attitude)

*C.c.* component coefficients (coefficient of the attitude in principal component)

## Discussion

CIT ECHO was developed to provide an opportunity for ongoing training in CIT best practices and to develop a network where LEOs have regular and frequent opportunities to communicate with mental health experts and other officers. While the original ECHO model and subsequent adaptations have been evaluated and shown to be helpful for participants (Peterson and Densley 2018; Watson et al. 2017), CIT ECHO is the first to use the ECHO model to train officers in CIT best practices. Overall, participants in our program reported increases in knowledge regarding mental illness and community mental health resources; increased self-efficacy in working with individuals with mental illness; and more compassionate attitudes towards individuals with mental illness. Each of these outcomes will be examined in turn below.

### Knowledge Increases

When controlling for factors including years of service, position, and exposure to multiple module topics, on average, respondents reported that the didactic component of CIT ECHO added “much” to their knowledge regarding a particular presentation topic. In particular, presentations that focused on Psychiatric Diagnosis and Substance Use appeared to be the most helpful to officers, suggesting that officers perceived themselves to derive the greatest benefit from instruction on different mental health and substance use disorders they may encounter in the field. Concerning knowledge of community mental health resources, all modules were rated to be helpful, though the modules Officer Self-Care and Special Training were rated as less helpful than the other modules as they did not focus on these types of resources.

The above findings make both intuitive and practical sense. Police contact of individuals with mental health issues is a common occurrence. A recent systematic review suggested that 25% of individuals with a mental illness have been arrested at some point in their lifetime, that 12% of individuals have encountered police in their pathway to mental health services, and that approximately one in every 100 police dispatches is coded as a primary mental health concern (Livingston 2016). Other research posits that somewhere between six and 20 percent of police contacts involve individuals with a serious mental illness (Watson et al. 2008). Despite this reality, police training in mental health and substance use disorders—though becoming more prevalent—is still relatively scant. In their 2020 national survey of police mental health training practices, Fiske et al. (2020) found that LEOs received only approximately 20-h of pre-service and roughly 17-h of in-service mental health-focused training. In short, given the frequency with which police officers may encounter individuals with mental health disorders in the field coupled with their limited training in mental illness; it comes as little surprise that participants in CIT ECHO found content on recognizing mental health symptoms to be particularly useful. Notably, this finding is also consistent with the extant research on general CIT training; for example, a recent systematic review of CIT outcome studies indicated that officers undergoing CIT training self-reported feeling more prepared for contact with individuals with mental illness as a result of their training, particularly as regards knowledge of mental health presentations and crisis intervention skills (Peterson and Densley 2018).

The finding that the Officer Self-Care and Special Training modules were least helpful in terms of enhancing knowledge of community mental health resources is not surprising given that the foci of these didactics were not on increasing awareness of resources in the community. The didactics within the Officer Self-Care Module focused on providing individual strategies (e.g., coping skills and meditation) and skills for LEOs to address stress and their own mental health challenges. Examples of didactics include Effective Coping Mechanisms, Stress Management, Mindfulness, Peer Support, and Tips for Happiness. Didactics within the Special Training module aimed to increase knowledge regarding specific police-related topics not captured within the other modules, such as The Role of Media in Reporting Mass Shootings, The Sequential Intercept Model (Munetz and Griffin 2006), Transgender 101, and Introduction to the Criminal Psychology of Serial Killers. It is important to note that the Officer Self-Care module was not included in the original curriculum for CIT ECHO. Shortly after CIT ECHO was implemented, there were several requests from officers to have presentations on topics related to self-care and stress management. This request is not surprising given that recent research suggests roughly a quarter of LEOs experience moderate health problems (either physical or mental health issues) and approximately 6% evidence broad health problems (Mumford et al. 2021).

### Self-Efficacy Increases

Regarding self-efficacy, several key findings emerged. First, when controlling for factors including years of service, position, and exposure to multiple module topics, on average, participants reported in the weekly survey that, as a result of the didactic, they were “very likely” to utilize information learned in their daily job duties. Here, the Psychiatric Diagnosis module was perceived to be significantly more helpful than most other modules in aiding LEOs’ daily duties. In contrast, the Special Training module was perceived to be less helpful than most other modules. Second, controlling for years of service, position, and exposure to multiple module topics, on average, LEOs reported in the weekly survey that as a result of the didactic, they felt “somewhat” efficacious in their ability to interact with an individual in a mental health crisis. Here, the Psychiatric Diagnosis module was again perceived to be more helpful than other modules.

The remaining intriguing findings regarding self-efficacy stem from the responses of officers who participated in at least three CIT ECHO didactic sessions. For officers in this group, there was a significant association between participation in CIT ECHO and comfort in dealing with individuals with mental illness, such that a vast majority (89.7%) of participants reported feeling comfortable dealing with individuals presenting with mental health issues, as opposed to reporting feeling either neutral or not comfortable. There was a significant association between participating in CIT ECHO and self-perceived ability to determine which individuals with mental illness should be diverted to a hospital/emergency room as opposed to taken to jail, such that nearly half of LEOs felt they could make this distinction as opposed to either feeling neutral towards or unable to make this differentiation. There was a significant association between participating in CIT ECHO and feeling comfortable utilizing verbal de-escalation techniques, such that over half of LEOs felt confident in their abilities as opposed to feeling neutral or apprehensive about their ability to use verbal de-escalation techniques. Finally, there was a significant association between participating in CIT ECHO and LEOs’ perception that they knew who to call for advice about interacting with individuals living with a mental illness, such that the vast majority (84.5%) of LEOs reported feeling they could identify resources to reach out to as opposed to feeling either neutral or hesitant in their ability to identify appropriate consultation resources.

Of particular interest is the observed association between position and two of the four statements that assessed self-efficacy. Uniformed LEOs were significantly more likely than non-uniformed LEOs to agree to the statement that, because of CIT ECHO, “They are able to determine if a person living with mental illness should be taken to jail or to a hospital/emergency room.” Uniformed LEOs compared to non-uniformed LEOs were also significantly more likely to agree with the statement, because of CIT ECHO, “I am able to utilize verbal de-escalation techniques effectively’ compared to non-uniformed LEOs.” Both of these skills are valuable to police officers in their interactions with individuals with mental illness and/or substance use disorders. The greater reported impact observed among uniformed LEOs compared to non-uniformed LEOs is not surprising given that non-uniformed LEOs are more senior than the uniformed LEOs and many in the sample were dedicated mental health detectives. Because of their years of experience and specialization, non-uniformed LEOs come to CIT ECHO already knowing the appropriate disposition for individuals with mental illness (i.e., treatment rather than incarceration) and how to utilize verbal de-escalation techniques.

Taken together, these findings indicate that officers participating in CIT ECHO evidenced increased confidence in their abilities to utilize CIT ECHO module content in their everyday job activities. Regarding the weekly survey, similar to the didactics’ impact on self-reported knowledge, the Psychiatric Diagnosis module also appeared most helpful to LEOs in terms of self-efficacy. Again, as above, given the frequency with which LEOs encounter individuals with mental health issues in the field—and given the lack of emphasis that LEO training has on mental health issues—this finding is unsurprising and intuitive. To effectively tackle an issue—such as appropriately interacting with an individual in a mental health crisis—the actor must first possess the necessary foundational knowledge to do so. As such, it follows that the module that might lead to the greatest feelings of self-efficacy in officers is the Psychiatric Diagnosis module, which focused on recognizing specific mental health presentations so that officers knew when to employ a specialized skill set for working with individuals living with mental health issues. In contrast, the Special Training module appeared to be the least helpful, as LEOs reported they felt significantly less likely to employ didactic content in their daily duties for this module as compared to the CIT Policing, De-Escalation and Communication Skills, Officer Self-Care, and Substance Use modules. As above, this finding also makes sense when considering module content.

### Attitude Shifts

In line with increases in knowledge of and confidence in working with individuals with mental health symptoms, participation in CIT ECHO also triggered a shift in LEOs’ attitudes regarding individuals with mental illness and criminal justice involvement. In retrospective reflection regarding their attitudes towards individuals with mental illness prior to participating in CIT ECHO and after participating in CIT ECHO, LEOs reported significant positive shifts in attitudes. Two of these shifts were notable in terms of endorsement of mental illness myths. Prior to CIT ECHO officers were more likely to perceive individuals with a mental illness to be perpetrators of crime/violence than the victims of crime/violence; however, after CIT ECHO, LEOs’ attitudes regarding these two ideas became much more ambivalent (i.e., a shift to feeling neutral about the likelihood that someone with mental illness would be either a victim or perpetrator of a crime/violence). This is particularly notable, as it reflects a more accurate perception of the connection between mental illness and criminality/violence; indeed, research suggests individuals with mental illness are more likely to be victims of violence/criminality than to perpetrate them (Desmarais et al. 2014; Maniglio 2009). Three other shifts involved perceptions of how LEOs should interact with individuals with mental illness. As above, these shifts reflected a move from disagreeing with a statement to feeling more neutral about it. After participating in CIT ECHO, instead of disagreeing that it was the job of LEOs to help link individuals with treatment to mental illness, LEOs felt more ambivalent. Additionally, instead of feeling that individuals with mental illness may not be responsive to de-escalation techniques, LEOs felt more neutral. Also, as opposed to feeling that individuals with mental illness required the use of force to maintain officer safety, LEOs felt more equivocal.

Furthermore, the results of PCA found an overall shift in attitudes among LEOs. Prior to participating in CIT ECHO, PCA revealed that officers’ primary sentiment towards individuals living with mental illness was one of paternalism. In short, LEOs felt a duty to help individuals with mental illness, because such individuals were at increased risk for victimization from violence and did not therefore require the use of force. Secondary sentiment revealed officers also had a stigma-like mindset towards individuals with mental illness, with LEOs disagreeing with the statement that people living with a mental illness are more likely to be a victim of a crime than to commit a crime. In contrast, post-CIT ECHO, the PCA revealed that officers still strongly believe that it is their job to link/connect people with mental illness to treatment but their attitudes shifted towards a greater understanding of the challenges experienced by individuals with mental illness (i.e., the likelihood of them being a victim of crime as well as violence), and their ability to respond to de-escalation techniques.

These shifts in attitudes are important, given the prevalence (as reviewed above) with which LEOs encounter individuals with mental illness in the deinstitutionalization era, as well as the increased chances of use of deadly force when officers encounter someone with mental illness (estimated to be as high as 16 times the rate for individuals without a mental illness) (Fuller et al. 2015). Though in the past it may not have been as pressing for LEOs to be properly equipped to work with individuals with mental illness—when many individuals with mental illness were institutionalized as opposed to being treated in the community—in recent times, LEOs are increasingly called to respond to mental health crisis situations (Barker 2013). Note that this is not to suggest that treating individuals with mental illness in an institution is indicated; rather, it is simply an observation that the expected duties of LEOs have changed as the mental health field’s ability to treat individuals in the community has advanced. To the extent that participation in CIT ECHO helped shift perception of LEOs that they were working on behalf of individuals with mental illness and should adjust their interactions appropriately from ideas that it is not a primary part of their role to respond to individuals with mental illness differently than individuals without mental illness but rather just a duty that is thrust upon them, that movement is certainly encouraging.

### Limitations and Future Research

While the results of the evaluation are important for informing the future implementation of CIT ECHO and its replication in other jurisdictions and states, our evaluation has several limitations. While the response rate was higher than observed in other evaluations of ECHO (Baker et al. 2020), the response rate for the follow-up survey was less than 50% (48.7%) and the response rate for the post-session survey was especially low (18.5%). The respondents may not be representative of all those LEOs who attend CIT ECHO. Furthermore, research on response bias has shown that those who respond tend to be more satisfied than those who do not respond to feedback surveys (Mazor et al. 2002). Thus, the results from this evaluation need to be interpreted with caution as they may be an overestimate of the impact of CIT ECHO. On the other hand, LEOs who participate in CIT ECHO are also a select group of officers, typically coming with a strong foundation, understanding, and interest in CIT (all of them completed the 40-h CIT training prior to joining CIT ECHO), so the impact of CIT ECHO may be slightly attenuated among this study sample. Another limitation is that we failed to have any information on attitudes, knowledge, and self-efficacy among participants prior to engaging in CIT ECHO. While the retrospective pre-post test design was used to measure changes in attitudes over time, and the strengths of this approach compared to a traditional pre-post assessment has been documented, results from this evaluation must be interpreted with caution (Bhanji et al. 2012; Geldhof et al. 2018; Thomas et al. 2019). In addition, the measurement of the impact of CIT ECHO on knowledge was limited to only two questions on the post-session survey and on attitudes was limited to questions on the follow-up survey only. Also, different questions were used to measure the impact of CIT ECHO on perceptions of self-efficacy on the post-session survey and follow-up survey. Future evaluations of CIT ECHO should include the same questions in both surveys to be able to draw more solid conclusions regarding the impact of CIT ECHO on knowledge, attitudes, and perceptions of self-efficacy. With respect to the use of two surveys, data were also collected at different time points, As a reminder, the median number of sessions attended was 1, with an interquartile range of 1–4 sessions and a range of 1–55 sessions. The number of CIT ECHO sessions completed was not controlled for in the analyses, and undoubtedly the impact of CIT ECHO would vary among LEOs who were “exposed” to CIT ECHO for the first time compared to those who had multiple exposures. Finally, all of the data collected in this evaluation were self-report, and self-reported data are subject to biases and limitations (Taylor et al. 1998). Given these limitations, and the promising results of this exploratory evaluation, a more scientifically rigorous evaluation is warranted.

Of note, a common observation of CIT outcome research is that has focused mainly officer-level outcomes––such as perceived increases in knowledge regarding mental health or self-efficacy in working with individuals with mental health issues—as opposed to community-level outcomes (aside from increased diversion of individuals to mental health services) (Peterson and Densley 2018; Rogers et al. 2019; Taheri 2016). This calls into question the usefulness of CIT programs at the community level—in other words, does CIT truly further positive community outcomes, such as increased officer and civilian safety and cost-effectiveness? Though this is also a limitation of the current study, our outcomes do create a reason for optimism for positive community outcomes when considered through the lens of the Theory of Planned Behavior (TPP) (Ajzen 1991, 2005). TPP posits that in predicting positive health-related changes, it is helpful to consider individuals’ attitudes towards a behavior; normative beliefs about a behavior; and perception of behavioral control. In short, positive behavior change might be expected when an individual’s attitudes towards a behavior are favorable; when societal norms towards a behavior are favorable (and, hence, one is motivated to engage in the behavior for social approval); and whether an individual is confident in his/her ability to engage in a behavior. This in turn influences an individual’s *intention* to engage in the behavior, and subsequent behavior change. The TPP has shown to be predictive of positive behavioral change in health domains, such as addressing addictive behaviors or exercise behavior (Godin and Kok 1996).

Here, our results indicate positive attitude shifts in LEOs, including a shift in beliefs regarding myths of mental illness and regarding LEOs’ roles in working with individuals with mental illness. Additionally, shifts in LEOs’ knowledge of mental illness symptoms and community resources to deal with them, as well as shifts in LEOs’ perceived ability to recognize mental health symptoms and make use of de-escalation techniques community resources. As such, because LEOs’ attitudes about and self-efficacy regarding working with individuals with mental illness shifted in a positive direction—and because it has become increasingly apparent in the USA that change is needed regarding how the criminal justice system works with individuals with mental illness—it is reasonable to believe that CIT ECHO has the *potential* to produce intention for change and positive community-level outcomes, particularly in providing continuous support for LEOs in CIT best practices. However, to truly address the limitations noted above, the CIT ECHO hub team is currently considering methodology for future evaluations that would allow examination of community-level outcomes, as well as differences on these community-level outcomes between officers who do and do not participate in CIT ECHO.

## Conclusion

Ongoing training in mental health for officers is both essential and lacking in law enforcement today. CIT ECHO offers one of the only models for continuous and constantly updated training for mental health encounters for police. The model has been shown here to improve self-efficacy and knowledge in law enforcement. CIT ECHO is available to law enforcement throughout the country, and other than a time commitment, is free for participants. There is no travel, and the mental health training is from psychiatrists and other experts in the field. The model is easily adapted to its audience and is scalable; there’s essentially no limit to the number of participants who can join the program. Most agencies that offer any advanced training in mental health management give a single 40-h course, without follow-up. Though this is an important model, classes are self-limited, and designed without structured continuing education. CIT ECHO offers continuing education, is flexible, and is easy to attend. CIT ECHO also offers a platform to help officers with their own mental health care. The training module on Officer Self-Care has been well received. Law enforcement has extremely stressful jobs, and CIT ECHO offers a way for them to connect to other officers, feel comfortable around clinicians, and learn not only about how to help the public better, but also about improving their own self-care. Finally, one of the early challenges with the delivery of CIT ECHO related to the use of the videoconferencing technology used for weekly sessions. Officers’ reported being intimated by the technology, which resulted in low attendance and hesitation in using the video capabilities of Zoom among those who did join the weekly sessions (16). With the widespread use of Zoom during the COVID-19 pandemic, we noticed that LEOs in NM became much more comfortable with using this technology for training, resulting in higher attendance and use of video. This recent shift in acceptability of Zoom technology is beneficial, in that it improves the likelihood of successful replication of CIT ECHO in other jurisdictions.

## Data Availability

Deidentified data presented in this study are available on request from the corresponding author. The data are not publicly available due to ethical reasons. The research protocol and data analysis syntax and codes may also be obtained from the corresponding author.

## References

[CR1] Ajzen I (1991). The theory of planned behavior. Organizational Behavior and Human Decision Processes.

[CR2] Ajzen I (2005). *Attitudes, personality and behavior* (2nd edition).

[CR3] Arora S, Kalishman S, Dion D, Som D, Thornton K, Bankhurst A, Boyle J, Harkins M, Moseley K, Murata G, Komaramy M, Katzman J, Colleran K, Deming P, Yutzy S (2011). Partnering urban academic medical centers and rural primary care clinicians to provide complex chronic disease care. Health Affairs (Project Hope).

[CR4] Arora S, Kalishman SG, Thornton KA, Komaromy MS, Katzman JG, Struminger BB, Rayburn WF (2017). Project ECHO: a telementoring network model for continuing professional development. J Contin Educ Health Prof.

[CR5] Bahora M, Hanafi S, Chien VH, Compton MT (2008). Preliminary evidence of effects of crisis intervention team training on self-efficacy and social distance. Administration and Policy in Mental Health.

[CR6] Baker RT, Casanova MP, Whitlock JN, Smith LH, Seegmiller JG (2020). Expanding access to health care: evaluating project Extension for Community Health Care Outcomes (ECHO) Idaho’s tele-education behavioral health program. J Rural Ment Health.

[CR7] Barker J (2013) Police encounters with the mentally ill after deinstitutionalization. 30(1). https://www.psychiatrictimes.com/view/police-encounters-mentally-ill-after-deinstitutionalization

[CR8] Bhanji F, Gottesman R, de Grave W, Steinert Y, Winer LR (2012). The retrospective pre-post: a practical method to evaluate learning from an educational program. Acad Emerg Med Off J Soc Acad Emerg Med.

[CR9] Bonfine N, Ritter C, Munetz MR (2014). Police officer perceptions of the impact of Crisis Intervention Team (CIT) programs. Int J Law Psychiatry.

[CR10] Compton MT, Bahora M, Watson AC, Oliva JR (2008). A comprehensive review of extant research on Crisis Intervention Team (CIT) programs. The Journal of the American Academy of Psychiatry and the Law.

[CR11] Compton MT, Chien VH (2008) Factors related to knowledge retention after crisis intervention team training for police officers. Psychiatr Serv (Washington, D.C.), 59(9):1049–1051. 10.1176/ps.2008.59.9.1049 10.1176/ps.2008.59.9.1049 18757600

[CR12] Compton MT, Demir Neubert BN, Broussard B, McGriff JA, Morgan R, Oliva JR (2011). Use of force preferences and perceived effectiveness of actions among Crisis Intervention Team (CIT) police officers and non-CIT officers in an escalating psychiatric crisis involving a subject with schizophrenia. Schizophrenia Bulletin.

[CR13] Compton MT, Esterberg ML, McGee R, Kotwicki RJ, Oliva JR (2006). Brief reports: crisis intervention team training: changes in knowledge, attitudes, and stigma related to schizophrenia. Psychiatric Services.

[CR14] Crisanti AS, Earheart JA, Rosenbaum NA, Tinney M, Duhigg DJ (2019). Beyond crisis intervention team (CIT) classroom training: videoconference continuing education for law enforcement. Int J Law Psychiatry.

[CR15] Cuddeback GS, Kurtz RA, Wilson AB, VanDeinse T, Burgin SE (2016). Segmented versus traditional crisis intervention team training. J Am Acad Psychiatry Law.

[CR16] Desmarais SL, Van Dorn RA, Johnson KL, Grimm KJ, Douglas KS, Swartz MS (2014). Community violence perpetration and victimization among adults with mental illnesses. Am J Public Health.

[CR17] Ellis HA (2014). Effects of a Crisis Intervention Team (CIT) training program upon police officers before and after Crisis Intervention Team training. Arch Psychiatr Nurs.

[CR18] Fiske ZR, Songer DM, Schriver JL (2020). A national survey of police mental health training. J Police Crim Psychol.

[CR19] Fuller DA, Lamb HR, Biasotti M, Snook J (2015) Overlooked in the undercounted (p. 27). Treatment Advocacy Center Office of Reserch & Public Affairs. https://www.treatmentadvocacycenter.org/storage/documents/overlooked-in-the-undercounted.pdf

[CR20] Geldhof GJ, Warner DA, Finders JK, Thogmartin AA, Clark A, Longway KA (2018). Revisiting the utility of retrospective pre-post designs: the need for mixed-method pilot data. Evaluation and Program Planning.

[CR21] Godin G, Kok G (1996). The theory of planned behavior: a review of its applications to health-related behaviors. American Journal of Health Promotion: AJHP.

[CR22] Kaushal K (2016). Response shift bias in pre- and post-test studies. Indian J Dermatol.

[CR23] Komaromy M, Bartlett J, Manis K, Arora S (2017). Enhanced primary care treatment of behavioral disorders with ECHO case-based learning. Psychiatric Services.

[CR24] Livingston JD (2016). Contact between police and people with mental disorders: a review of rates. Psychiatr Serv.

[CR25] Maniglio R (2009). Severe mental illness and criminal victimization: a systematic review. Acta Psychiatr Scand.

[CR26] Mazor KM, Clauser BE, Field T, Yood RA, Gurwitz JH (2002). A demonstration of the impact of response bias on the results of patient satisfaction surveys. Health Serv Res.

[CR27] Mumford EA, Liu W, Taylor BG (2021) Profiles of U.S. Law Enforcement Officers’ physical, psychological, and behavioral health: results from a nationally representative survey of officers. Police Q 24(3):357–381. 10.1177/1098611121991111

[CR28] Munetz MR, Griffin PA (2006) Use of the Sequential Intercept Model as an approach to decriminalization of people with serious mental illness. Psychiatr Serv (Washington, D.C.), 57(4):544–549. 10.1176/ps.2006.57.4.544 10.1176/ps.2006.57.4.544 16603751

[CR29] Peterson J, Densley J (2018). Is Crisis Intervention Team (CIT) training evidence-based practice? A systematic review. J Crime Justice.

[CR30] Plotkin M, Peckerman T (2017) The variability in law enforcement state standards: a 42-state survey on mental health and crisis de-escalation training. The Council of State Governments Justice Center. https://csgjusticecenter.org/wp-content/uploads/2017/02/JC-LE-Survey.pdf

[CR31] Rogers MS, McNiel DE, Binder RL (2019) Effectiveness of police crisis intervention training programs. J Am Acad Psychiatry Law, 47(4):414–421. 10.29158/JAAPL.003863-19 10.29158/JAAPL.003863-19 31551327

[CR32] Skeem J, Bibeau L (2008). How does violence potential relate to crisis intervention team responses to emergencies?. Psychiatr Serv.

[CR33] Taheri SA (2016). Do crisis intervention teams reduce arrests and improve officer safety? a systematic review and meta-analysis. Crim Justice Policy Rev.

[CR34] Taylor PJ, Lamers A, Vincent MP, O’Driscoll MP (1998). The validity of immediate and delayed self-reports in training evaluation: an exploratory field study. Appl Psychol.

[CR35] Teller JLS, Munetz MR, Gil KM, Ritter C (2006). Crisis intervention team training for police officers responding to mental disturbance calls. Psychiatr Serv.

[CR36] Thomas EV, Wells R, Baumann SD, Graybill E, Roach A, Truscott SD, Crenshaw M, Crimmins D (2019). Comparing traditional versus retrospective pre-/post-assessment in an interdisciplinary leadership training program. Matern Child Health J.

[CR37] Watson AC, Compton MT, Draine JN (2017). The crisis intervention team (CIT) model: an evidence-based policing practice?. Behav Sci Law.

[CR38] Watson AC, Fulambarker AJ (2012). The crisis intervention team model of police response to mental health crises: a primer for mental health practitioners. Best Pract Ment Health.

[CR39] Watson AC, Morabito MS, Draine J, Ottati V (2008). Improving police response to persons with mental illness: a multi-level conceptualization of CIT. Int J Law Psychiatry.

[CR40] Watson AC, Ottati VC, Morabito M, Draine J, Kerr AN, Angell B (2010). Outcomes of police contacts with persons with mental illness: the impact of CIT. Adm Policy Ment Health.

[CR41] Watson AC, Wood JD (2017). Everyday police work during mental health encounters: a study of call resolutions in Chicago and their implications for diversion. Behav Sci Law.

